# The Discovery of the Buffer Capacity of Various Types of Polyelectrolyte Microcapsules

**DOI:** 10.3390/polym13224026

**Published:** 2021-11-21

**Authors:** Alexey V. Dubrovskii, Aleksandr L. Kim, Egor V. Musin, Bulat R. Ramazanov, Sergey A. Tikhonenko

**Affiliations:** 1 Institute of Theoretical and Experimental Biophysics Russian Academy of Science, Institutskaya st., 3, 142290 Puschino, Russia; dav198@mail.ru (A.V.D.); kimerzent@gmail.com (A.L.K.); eglork@gmail.com (E.V.M.); 2 Faculty of Biology, M. V. Lomonosov Moscow State University, 119991 Moscow, Russia; bulat2398@gmail.com

**Keywords:** polyelectrolyte microcapsules, microcapsules, buffer capacity, polyelectrolyte

## Abstract

Polyelectrolyte microcapsules, which are obtained by the method of alternate adsorption of oppositely charged polyelectrolytes onto colloidal particles of micron size, are widely used in science and industry. Nevertheless, the properties of microcapsules are still poorly understood. In particular, there is no information in the literature on the buffer capacity. However, information on the presence of a buffer capacity and an understanding of its mechanisms can both simplify the use of microcapsules and expand the scope of their application. In this regard, the buffer capacity of various types of microcapsules was studied. It was found that polyelectrolyte microcapsules consisting of polyallylamine, and polystyrene sulfonate have a buffer capacity. In addition, in an acidic medium, the buffer capacity of microcapsules containing BSA is significantly greater than that of microcapsules without protein. This is due to the fact that BSA contributes to the buffering of microcapsules. Differences in the behaviour of the buffer capacity of microcapsules with the composition (PAH/PSS)_3_ and (PSS/PAH)_3_ were found. In addition, a hypothesis has been proposed that regions of unbound polyallylamine are responsible for the buffering properties of polyelectrolyte microcapsules. This hypothesis is confirmed by the fact that incubation of microcapsules in 0.5 M NaCl increases the amount of unbound polyallylamine, which leads to an increase in the buffer capacity of microcapsules at alkaline pH values higher than the buffer capacity of capsules in an aqueous solution.

## 1. Introduction

Polyelectrolytes (PE) are polymers whose monomers have groups capable of ionization in solution. This electrostatic property affects many of the macromolecular characteristics of PE such as viscosity, chain shape, and energy transfer parameters. These features of polyelectrolytes make it possible to use them as destabilizers of colloidal systems and initiators of flocculation in oil production and surface water treatment. In addition, they are frequently used as thickeners, emulsifiers, conditioners, clarifiers and even flow resistance reducers in medicine, food, textile, and other industries [1]. One of the significant and frequently used properties of polyelectrolytes is their buffer capacity.

The buffer capacity (BC) of a solution is the ability of a solution to maintain a constant pH value when an acid or alkali is added. High buffering capacity is typical for weak polyelectrolytes, which have a dissociation constant (pKa or pKb) in the pH range from ~2 to ~10. They are characterized by a wide range of buffer capacities because these PEs change the degree of their (de) protonation and charge density when the pH of the solution changes. Thus, the high buffering capacity of the polyelectrolyte can be used to create the “proton sponge” effect [2], for example, in lysosomes/endosomes and stimulate the release of drugs [3] or stimulate the death of bacteria [4]. In addition, polyelectrolytes can maintain a stable pH value during the cultivation of bacterial and eukaryotic cells [5].

The study of the buffer properties of polyelectrolyte complexes (PC) is a logical continuation of this direction. In particular, it has been shown [6,7,8] that the pH buffering activity of LbL assemblies [9] of weak and strong polyelectrolytes protects metal surfaces from corrosion. In addition, the buffer capacity of PC can be controlled by changing the polyelectrolyte composition, as shown by DV Andreeva et al., who used chitosan as a surface pH buffer to protect supramolecular fibers triaminopyrimidine (TAP) and cyanuric acid modified with a hexanoic acid side chain (CyCo6) from pH-mediated degradation [10]. However, the buffer capacity of the polyelectrolyte complex is not only protection against the acidity of the medium, but also an important parameter of diagnostic systems, which must be taken into account in order to obtain reliable results. For example, Nedal Y. Abu-Thabit developed a pH-sensitive sensor whose membrane consisted of a polyelectrolyte complex and increased the response time of the system [11].

The buffer capacity of the polyelectrolyte complex mainly depends on the buffer capacity of the weak polyelectrolyte in the PC composition [12]. Polyelectrolytes with weak (de) protonating groups, such as carboxyls and amines, absorb or release protons (depending on pH) during complex formation. This leads to a change in pH, but any polyelectrolyte not included in the complex will act as a buffer and increase the buffer capacity of the solution [13]. In addition, the different chemical environment, and steric properties of the polyelectrolyte lead to a range of different pKa values for the same monomer at different positions of the polyelectrolyte. For example, Richard et al. investigated the buffering capacity of linear and branched polyethyleneimine, which had the same molecular weight and the same number of charged groups [14]. It was shown that these polyelectrolytes, both free and as part of the complex, had different values of the buffer capacity in different pH ranges. Thus, the complex structure of polyelectrolyte supramolecular systems, which have a complex spatial organization and chemical environment, can have their own behaviour of changing the buffer capacity.

One of the examples of polyelectrolyte supramolecular systems are polyelectrolyte microcapsules (PMC). PMC are spherical containers, which are obtained by the method of alternate adsorption of oppositely charged polyelectrolytes onto colloidal particles of micron size. Currently, research is underway to create targeted delivery systems, diagnostic and theranostic systems based on polyelectrolyte microcapsules [15,16,17,18,19]. Nevertheless, the physicochemical properties of this object are still poorly understood, in particular, there is no information in the literature on the buffer capacity. This parameter is necessary for the encapsulation of pH-sensitive compounds, both for creating a buffer barrier for the encapsulated substance (metals, enzymes, polyelectrolytes, drugs) and for the correct interpretation of the results obtained when working with this object. For example, in the works of Antipina, M.N. et al. [20] and Kazakova, L.I. et al. [21] proposed the use of encapsulated pH-sensitive fluorescent labels (Amplex Red and SNARF-1) to create diagnostic systems. These dyes can change their own fluorescence depending on the pH of the solution, but the presence of the buffer capacity of the polyelectrolyte microcapsule can distort the received signals.

Thus, the purpose of our work is to discover and study the buffer capacity behaviour of different types of polyelectrolyte microcapsules at the pH range from 4 to 9.

## 2. Materials and Methods

### 2.1. Materials

Polyelectrolytes polystyrenesulfonate sodium (PSS) and polyallylamine hydrochloride (PAH) with a molecular mass of 70 kDa, bovine serum albumin (BSA), Ethylenediaminetetraacetic acid disodium salt dihydrate (EDTA) purchased in Sigma (St. Louis, MS, USA), sodium chloride, ammonium sulfate, sodium carbonate, calcium chloride from “Reahim”.

### 2.2. Preparation of CaCO_3_ Microspherulites

At stirring of 0.33 M Na_2_CO_3_, the 0.33 M CaCl^2^ was added (which contained 3 mg/mL of BSA if it was necessary). The stirring time was 30 s. The suspension was maintained until complete precipitation of the formed particles. The process of “ripening” of microspherolites was controlled with the help of a light microscope. Then, the supernatant was decanted, the precipitate was washed with water and used to prepare PMC. The microparticles were obtained with an average diameter of 4.5 ± 1 μm.

### 2.3. Preparation of Polyelectrolyte Microcapsules

The “BSA-free” type of polyelectrolyte microcapsules was obtained by layer-by-layer adsorbing the negatively or positively charged polyelectrolytes onto CaCO_3_ microspherulites, followed by dissolution of CaCO_3_. At the moment of dissolution of CaCO_3_ core the inner space of PMC is filled by interpolyelectrolyte complex [22]. Layer-by-layer adsorption of PAH and PSS on the CaCO_3_ microspherulites surface was carried out in polyelectrolytes solutions (concentration 2 mg/mL + 0.5 M NaCl). After each adsorption the CaCO_3_ particles with adsorbed polyelectrolytes were triple washed with a 0.5 M NaCl solution, which was necessary to remove unabsorbed polymer molecules. The particles were separated from the supernatant by centrifugation. After applying the required number of layers, the carbonate kernels were dissolved in a 0.2 M EDTA solution for 12 h. The resulting capsules were washed three times with water to remove core decay products. In the case of the PMC with an encapsulated BSA, we used a protein filled CaCO_3_ core which is dissolved at the final stage of preparation. The microcapsules were obtained with an average diameter of 4.5 ± 1 μm. The size and surface charge of microcapsules was measured using the dynamic light scattering method on a Zetasizer nano ZS device (Malvern, UK). The surface charge of PMC with interpolyelectrolyte complex of the following composition: (PAH/PSS)_3_—+23 ± 2.1 mV; (PSS/PAH)_3_—+22 ± 3.2 mV. The surface charge of PMC with protein of the following composition: (PAH/PSS)_3_–BSA—+23 ± 2.1 mV; (PSS/PAH)_3_–BSA—+21 ± 1.8 mV.

### 2.4. Measurement of Buffering Capacity

A suspension of microcapsules (PSS/PAH)_3_ (6.6 × 10^9^ microcapsules in 8 mL of water) was titrated with acid or base solutions in the pH range from 4 to 9. Titration was carried out by manual measurement. The acid or alkali (with a concentration of 0.001 M or 0.005 M) was added to the solution to change the pH of a solution by 0.02 or more. Buffering capacity was calculated from Equation (1) after estimating the slope of the titration curves at each point by the variation of the pH between previous and subsequent injections [14]:

(1) $$\mathrm{BC}=\frac{n_{\mathrm{NaOH}}\left(i+1\right)-n_{\mathrm{NaOH}}\left(i-1\right)\;}{\mathrm{pH}\left(i+1\right)-\;\mathrm{pH}\left(i-1\right)}$$

## 3. Results

**Figure 1:**
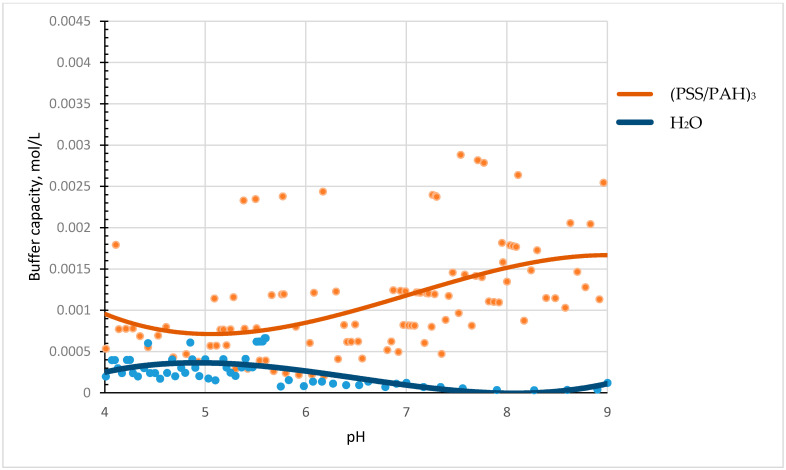
Buffer capacity of microcapsules (PSS/PAH)_3_ and water in the pH range from 4 to 9.

A suspension of microcapsules (PSS/PAH)_3_ (6.6 × 10^9^ microcapsules in 8 mL of water) was titrated with acid or alkali solutions in the pH range from 4 to 9 to reveal the presence of a buffer capacity of polyelectrolyte microcapsules. The experimental results are shown in Figure 1.

The figure shows that microcapsules have a buffer capacity (orange curve) that differs from the buffer capacity of water (blue curve).

**Figure 2:**
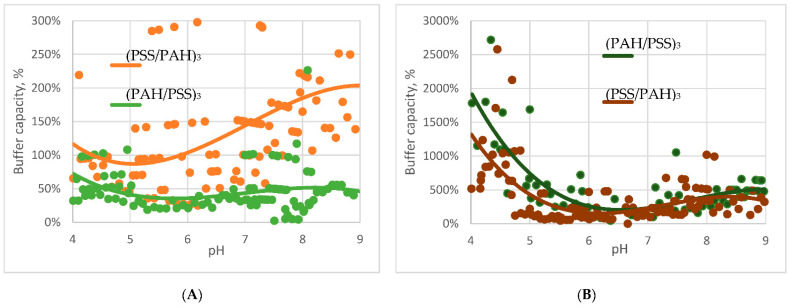
Buffer capacity of microcapsules (**A**) and microcapsules with encapsulated BSA (**B**).

The next stage of the study was to compare the behaviour of the buffer capacity of different types of PMC. The capacity of capsules of the following composition was investigated: (PSS/PAH)_3_, (PAH/PSS)_3_, (PSS/PAH)_3_–BSA and (PAH/PSS)_3_–BSA. Since PMCs are complex supramolecular systems, we cannot compare their buffer capacities directly in numerical values. In this regard, we normalized the values of the buffer capacities of microcapsules, where the value of the buffer capacity at pH 7 is 100%. The results of this study are shown in Figure 2.

The Figure 2A shows that microcapsules composition (PSS/PAH)_3_ have a higher buffering capacity than (PAH/PSS)_3_ and it increases in pH range from 6 to 9. The behaviour of buffer capacity of BSA-containing microcapsules in an acidic medium is higher (by about an order of magnitude) than the capacity of the protein-free capsules (At ph 4 the buffer capacity of (PSS/PAH)_3_ without BSA is 0.535332 mmol/L and with BSA is 2.682547 mmol/L). This effect is explained by the fact that in an acidic medium BSA has a high buffering capacity [23], which contributes to the total buffering capacity of the capsules. The higher buffer capacity of microcapsules ((PAH/PSS)_3_–BSA) (Figure 2B) is related to the fact that the encapsulated protein in such microcapsules at acidic pH values distributed in the interior of the capsule [24], as the result it makes a large contribution to the buffer properties of the capsules.

**Figure 3:**
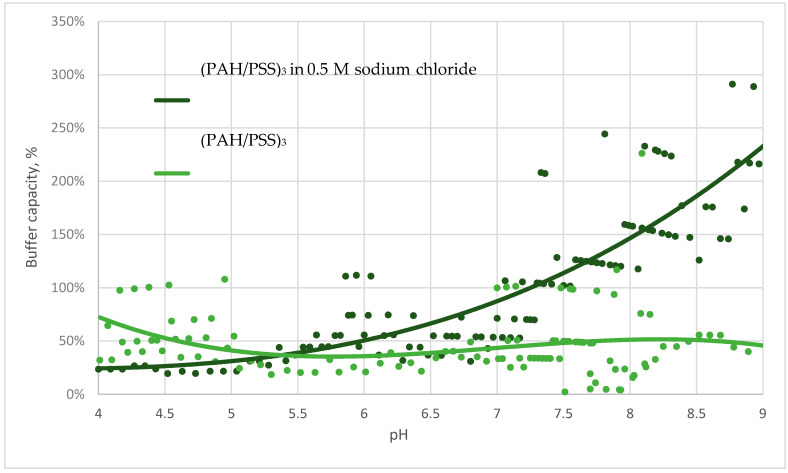
Buffer capacity of microcapsules (PAH/PSS)_3_ in water and sodium chloride solution in the pH range from 4 to 9.

The next step in our study was to determine the causes of the buffer capacity of microcapsules. We hypothesized that the main contribution to the buffer capacity of the protein-free PMC is made by the regions of free PAH since the microcapsule is a supramolecular system, which consists of both interpolyelectrolyte complexes (PAH and PSS) and unbound fragments of polyelectrolyte chains. To confirm this hypothesis, we studied the behaviour of the buffer capacities of microcapsules in a 0.5 M sodium chloride solution. The results of this study are shown in Figure 3.

The figure shows that the buffer capacity of microcapsules in salt increases in an alkaline medium. This confirms the hypothesis that the contribution to the buffer capacity is made by unbound PAH regions [25], the number of which strongly increases in the presence of salt [26].

**Figure 4:**
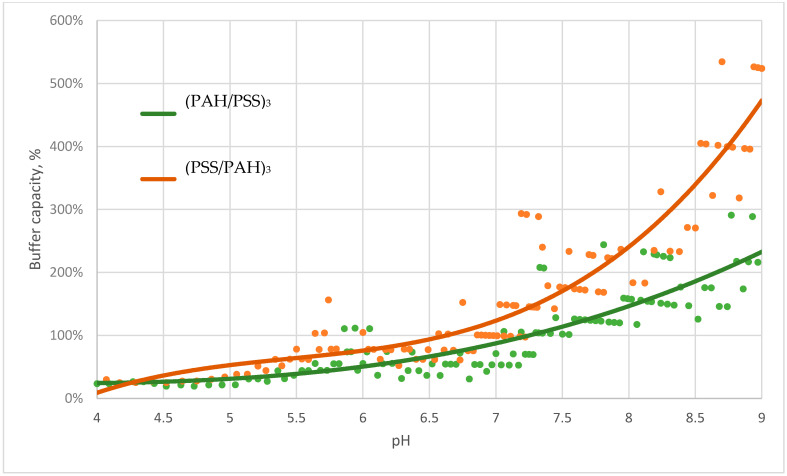
Buffer capacity of microcapsules (PAH/PSS)_3_ and (PSS/PAH)_3_ in sodium chloride in the pH range from 4 to 9.

This hypothesis is also confirmed by comparing the curves of the buffer capacity of microcapsules with the composition of microcapsules (PAH/PSS)_3_ and (PSS/PAH)_3_ in salt. The data are presented in Figure 4.

The figure shows that the buffer capacity of microcapsules with the upper layer of PAH in the alkaline pH range is higher, which is explained by the larger amount of unbonded region of PAH.

## 4. Conclusions

We titrated a suspension of polyelectrolyte microcapsules (PMC) consisting of polyalliamine (PAH) and polystyrene sulfonate (PSS) with acid and alkali solutions in the pH range from 4 to 9 to discover the buffer capacity of various types of microcapsules. It was found that microcapsules have a buffer capacity that differs from the buffer capacity of water and gradually increases from pH 5.5 to pH 9. A comparison of the buffer capacities of protein-containing and protein-free microcapsules showed that the buffer capacity of microcapsules with BSA is much higher in acidic pH values, which is explained by the significant contribution of protein to the total buffer capacity of such microcapsules in this range. Furthermore, differences in the behaviour of the buffer capacity of microcapsules with the composition (PAH/PSS)_3_ and (PSS/PAH)_3_ were found. In addition, we put forward the hypothesis of the presence of a buffer capacity of PMC, which consists of the fact that the regions of unbound weak PAH polyelectrolyte, which are contained in microcapsules, are responsible for the buffer properties of the capsules. To confirm this, we titrated PMC in a 0.5 M solution of sodium chloride, the presence of which leads to an increase in the amount of unpaired PAH. The results of this experiment showed that the buffer capacity of microcapsules in salt was significantly higher than their buffer capacity in water, which confirms our hypothesis.

## Data Availability

Data sharing not applicable.
